# Lysosome-Bursting Calcium Carbonate Nanoparticles Amplify Immunogenic Cell Death via Synergistic Phototherapy and Calcium Overload for Immune Checkpoint Blockade

**DOI:** 10.34133/research.1444

**Published:** 2026-10-12

**Authors:** Feifei Cui, Qifan Wu, Renjie Li, Mengfan Han, Chaokun Wang, Dailin Feng, Yingrui Su, Yuling Liu, Meng Meng, Jun Ye

**Affiliations:** ^1^ State Key Laboratory of Medicinal Chemical Biology, College of Pharmacy and KLMDASR of Tianjin, Nankai University, Tianjin, China.; ^2^ State Key Laboratory of Bioactive Substance and Function of Natural Medicines, Institute of Materia Medica, Chinese Academy of Medical Sciences & Peking Union Medical College, Beijing, China.; ^3^ Beijing Key Laboratory of Key Technologies for Natural Drug Delivery and Novel Formulations, Institute of Materia Medica, Chinese Academy of Medical Sciences & Peking Union Medical College, Beijing, China.

## Abstract

Immunogenic cell death (ICD) induction is a promising strategy to enhance cancer immunotherapy, yet its efficacy is often limited by insufficient immune activation. Herein, we develop a lysosome-bursting calcium carbonate nanoplatform (cRGD/MD@ICN) co-loaded with the photosensitizer indocyanine green (ICG) and a PD-L1 inhibitory peptide (^D^PPA-1) for synergistic phototherapy and calcium overload to amplify ICD and potentiate immune checkpoint blockade. The nanoparticle is decorated with cRGD for active tumor targeting and a matrix metalloproteinase-2 (MMP-2)-cleavable linker (MD) for on-demand release of ^D^PPA-1 in the tumor microenvironment. Upon endocytosis into tumor cells, the acidic lysosomal environment triggers rapid dissolution of the calcium carbonate core, releasing ICG and Ca^2+^, leading to lysosome bursting and subsequent calcium overload. Combined with near-infrared (NIR) irradiation, ICG-mediated phototherapy and calcium overload synergistically induce robust ICD, characterized by enhanced calreticulin exposure, HMGB1 release, and ATP secretion. In vivo studies demonstrate that cRGD/MD@ICN significantly suppresses orthotopic breast tumor growth and pulmonary metastasis by promoting dendritic cell maturation and intratumoral infiltration of CD4^+^ and CD8^+^ T cells. This lysosome-bursting nanoplatform offers a promising strategy to amplify ICD through synergistic phototherapy and calcium overload, thereby boosting the efficacy of immune checkpoint blockade for cancer treatment.

## Introduction

Immunogenic cell death (ICD) has gained substantial attention in cancer therapy by eliciting adaptive antitumor immunity [1–3]. During the ICD process, damaged or dying tumor cells release damage-associated molecular patterns (DAMPs), such as calreticulin (CRT), high-mobility group box 1 (HMGB1), and adenosine triphosphate (ATP), to promote dendritic cell (DC) maturation and initiate antigen-specific T cell responses, thereby developing systemic immune memory [4,5]. In recent years, phototherapeutic treatments, mainly including photodynamic therapy (PDT) and photothermal therapy (PTT), have emerged as advantageous means to promote ICD more precisely in a noninvasive way [6–8]. As reported, the U.S. Food and Drug Administration (FDA)-approved NIR dye indocyanine green (ICG) can facilitate PTT and PDT therapy, and provoke antitumor immune responses by inducing ICD responses [9,10]. However, the immune activation achieved by phototherapy alone is often limited and may be insufficient to fully suppress tumor progression or prevent recurrence [11,12]. Consequently, it is imperative to devise approaches that boost ICD efficacy, thereby potentiating the antitumor immune responses induced by phototherapy.

In enhancing anticancer immunity, immune checkpoint blockade (ICB) therapy has achieved remarkable success in clinical oncology [13,14]. As monoclonal antibodies targeting the PD-1/PD-L1 axis face challenges such as strong immunogenicity and poor tumor penetration [15,16], peptide-based checkpoint inhibitors (e.g., ^D^PPA-1) offer advantages in high binding affinity, low immunogenicity, and favorable tissue penetration [17,18]. Nevertheless, their susceptibility to enzymatic degradation in vivo and short plasma half-life significantly hinder the future applications [19]. Therefore, the development of co-delivery nanocarriers in loading the peptides and phototherapeutic agents has become a promising strategy to improve therapeutic outcomes.

As the nanotechnologies advance, calcium carbonate nanoparticles have emerged as an attractive drug delivery platform owing to excellent biocompatibility, biodegradability, and pH-responsive behavior [20,21]. More importantly, upon endocytosis by tumor cells, calcium carbonate nanoparticles rapidly dissolve in the acidic lysosomal environment, releasing calcium ions (Ca^2+^) and leading to increased lysosomal membrane permeability and even lysosomal rupture—a “lysosome-bursting” property that disrupts intracellular calcium homeostasis and induces calcium overload. Studies have revealed that sustained elevation of cytoplasmic Ca^2+^ impairs mitochondrial function, induces endoplasmic reticulum stress, and subsequently promotes DAMP release, thereby significantly amplifying ICD effects [22–24]. Up to now, the studies on calcium carbonate-mediated ICG delivery remain limited. Most existing work is focused on antitumor phototherapy toward primary tumor, while the application in ICB has not yet been investigated. Therefore, we hypothesize that leveraging the lysosome-bursting capability of calcium carbonate nanoparticles in synergy with phototherapy can achieve dual ICD amplification through combined calcium overload and phototherapeutic effects, ultimately enhancing the efficacy of ICB therapy.

Based on this rationale, we herein constructed a multifunctional nanoparticle system (cRGD/MD@ICN) (Fig. 1) that integrates cascade amplification of antitumor immunity by phototherapy and calcium overload with ICB treatment. This nanoplatform is constructed with a pH-responsive calcium carbonate core loaded with the photosensitizer ICG, coated with a lipid bilayer, and surface-conjugated with the immune checkpoint inhibitory peptide ^D^PPA-1. ^D^PPA-1 is a D-peptide that can effectively bind PD-L1 with high affinity in vitro and efficiently inhibit PD-1/PD-L1 interaction in vivo. In this nanoparticle, ^D^PPA-1 (D) is anchored to the lipid surface via a matrix metalloproteinase-2 (MMP-2)-cleavable linker peptide (M, PLGLAG), together presented as the “MD” fragment. Within the tumor microenvironment, overexpressed MMP-2 cleaves the MD peptide, exposing ^D^PPA-1 to block the PD-1/PD-L1 pathway [25,26]. Meanwhile, cRGD peptides on the nanoparticle surface specifically bind to integrin α_v_β_3_ overexpressed on tumor cells and tumor-associated vascular endothelium, thereby promoting tumor accumulation [27,28]. Following endocytosis into lysosomes, the acidic environment (pH < 5.5) triggers dissolution of the calcium carbonate core, releasing ICG and Ca^2+^, leading to lysosome bursting and intracellular calcium overload. Upon NIR irradiation, the phototherapeutic effects of ICG synergize with calcium overload to significantly amplify the ICD process. The design concepts of the nanosystem are demonstrated step-by-step, and antitumor immune effects are systematically evaluated in orthotopic and lung metastases of mouse model with breast cancer. This work presents a combined phototherapy with Ca^2+^ overload in enhancing ICD for ICB of cancer treatment, and offers a promising nanotherapeutic strategy to improve cancer immunotherapy for primary and pulmonary metastasis of breast cancer. Unlike conventional approaches that simply combine phototherapy with immune checkpoint inhibitors, our strategy leverages the unique “lysosome-bursting” property of calcium carbonate nanoparticles to achieve spatiotemporal coordination of phototherapy, calcium overload, and checkpoint blockade within a single nanoplatform—triggered in a programmed manner by the acidic lysosomal environment (for Ca^2+^ release and ICG liberation) and the tumor-overexpressed MMP-2 (for ^D^PPA-1 exposure), respectively.

**Fig. 1.**
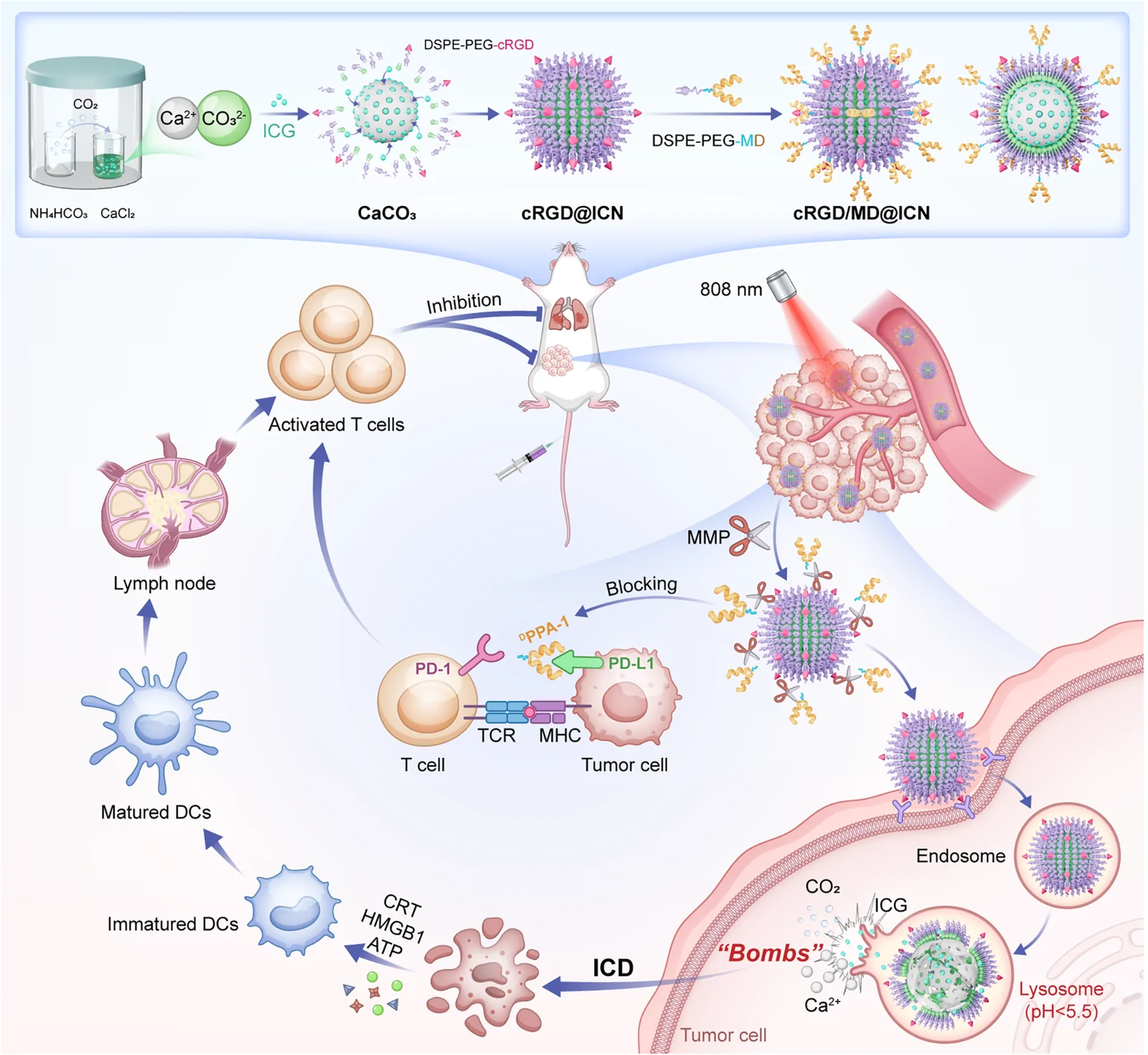
A multifunctional ICG nanoparticle system (cRGD/MD@ICN) to enhance antitumor immunity via phototherapy, calcium overload, and immune checkpoint treatment. The pH-responsive calcium carbonate nanoparticle is loaded with ICG and then attached with immune checkpoint inhibitory peptide ^D^PPA-1 on the surface. A cleavable peptide linker (M) of MMP-2 is used to connect ^D^PPA-1 (D). The MD fragment thus indicates MMP-2-sensitive peptide (M) linked with the ^D^PPA-1 peptide (D). In the tumor site, the MD peptide will be cleaved to expose ^D^PPA-1 for anticancer immunotherapy, and cRGD on the nanoparticle surface would promote tumor accumulation. In lysosome in tumor (pH < 5.5), the nanoparticles are disassociated, causing ICG phototherapy along with elevated Ca^2+^ to amplify the ICD process to achieve promoted anticancer immunity.

## Results and Discussion

### Structure confirmation of DSPE-PEG2000-MD

1,2-Distearoyl-sn-glycero-3-phosphoethanolamine (DSPE)-poly(ethylene glycol) 2000 (PEG2000)-MD was synthesized through a Michael addition reaction between the free sulfhydryl group of MD peptide and the maleimide moiety of DSPE-PEG-Mal, forming a stable thioether linkage at pH 6.5. Matrix-assisted laser desorption/ionization–time-of-flight (MALDI-TOF) analysis (Fig. S1) revealed that the main peak mass/charge ratio (*m*/*z*) value of DSPE-PEG2000-MD increased by approximately 2,153 compared to that of DSPE-PEG2000-Mal, which aligns with the theoretical molecular weight of the MD peptide segment (2,153 Da). In the ^1^H nuclear magnetic resonance (NMR) spectrum of DSPE-PEG2000-Mal (Fig. S2), the characteristic signal of the maleimide group was observed at around 6.9 ppm [29]. After conjugation with MD, this maleimide peak disappeared in the ^1^H NMR spectrum of DSPE-PEG2000-MD. These results confirmed successful synthesis of DSPE-PEG2000-MD.

### Characterization and pH-responsive release of the nanoparticles

We prepared ICG-loaded calcium carbonate nanoparticles (ICNs) via a gas diffusion reaction. Based on results of particle sizes and encapsulation efficiency obtained from standard calibration curve (Fig. S3), the ICG amount used in ICN preparation was optimized. When the loading amount of ICG was 1 mg, the encapsulation efficiency reached 98.93% ± 0.74%, and reproducibility was excellent (Table S1). The obtained ICN was modified, yielding different types of lipid-loaded ICN: PEG@ICN, cRGD@ICN, and cRGD/MD@ICN. Transmission electron microscopy (TEM) images revealed that ICN, PEG@ICN, and cRGD@ICN were spherical, whereas cRGD/MD@ICN displayed an irregular shape, likely attributable to surface coverage by peptides (Fig. 2A). All nanoparticles showed a uniform size distribution in dynamic light scattering analysis (Fig. 2B). The native ICN was positively charged due to abundant Ca^2+^, while phospholipid modification switched the surface to negatively charged (Fig. 2C). Elemental mapping (Fig. 2D) confirmed the presence of calcium element in all nanoparticles, and the existence of phosphorus element in PEG@ICN, cRGD@ICN, and cRGD/MD@ICN. X-ray photoelectron spectroscopy (Fig. 2E) analysis revealed characteristic Ca 2p (346.0 to 347.5 eV) and P 2p (132.5 to 134.0 eV) binding energies, further demonstrating successful modification of the phospholipids. After storage for 7 d at 4 °C, the particle size and zeta potential for all nanoparticles remained highly stable (Fig. 2F).

**Fig. 2.**
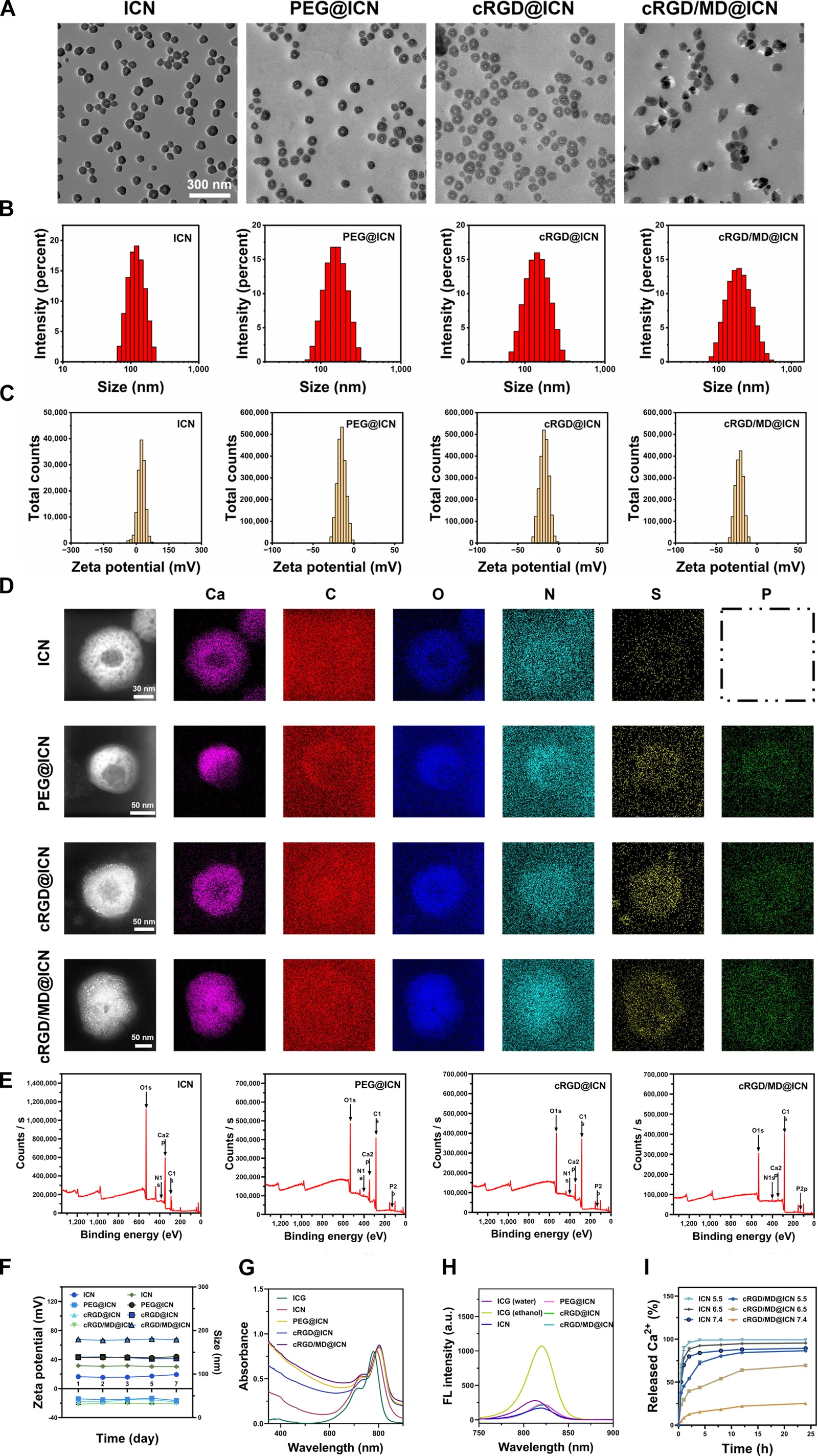
Characterizations of the ICG formulations and pH-responsive Ca^2+^ release from the proposed nanosystems. (A) TEM images, (B) particle size, and (C) zeta potential of different ICN formulations. (D) Element mappings and (E) x-ray photoelectron spectroscopy (XPS) of ICN samples. (F) Particle size and zeta potential of ICN samples after storage at 4 °C for 7 d. (G) UV absorbance spectra for each ICG formulation. (H) Fluorescence spectra of ICG in water or ethanol and different ICN samples. (I) pH-responsive Ca^2+^ release from ICN and cRGD/MD@ICN [*n* = 3, mean ± standard deviation (SD)].

Ultraviolet–visible (UV–vis) spectroscopy (Fig. 2G) showed that the characteristic absorption peak of ICG (784 nm) was red-shifted to 791 nm in ICN, and further to 803 nm after phospholipid modification, and all nanoparticles retained strong NIR absorption. All formulations exhibited strong NIR fluorescence in aqueous solution (Fig. 2H). The pH-responsive release of Ca^2+^ from cRGD/MD@ICN was then evaluated. As shown in Fig. 2I, the system remained stable at pH 7.4 within 24 h (percentage of released Ca^2+^ ≈ 25%) but almost dissociated at pH 5.5 (percentage of released Ca^2+^ ≈ 86%), demonstrating acid-responsive behavior of cRGD/MD@ICN. The ICG release profile could not be accurately determined due to its rapid degradation in the release medium; however, the stability of encapsulated ICG (Fig. 3A) supports its concomitant release with nanoparticle dissolution.

**Fig. 3.**
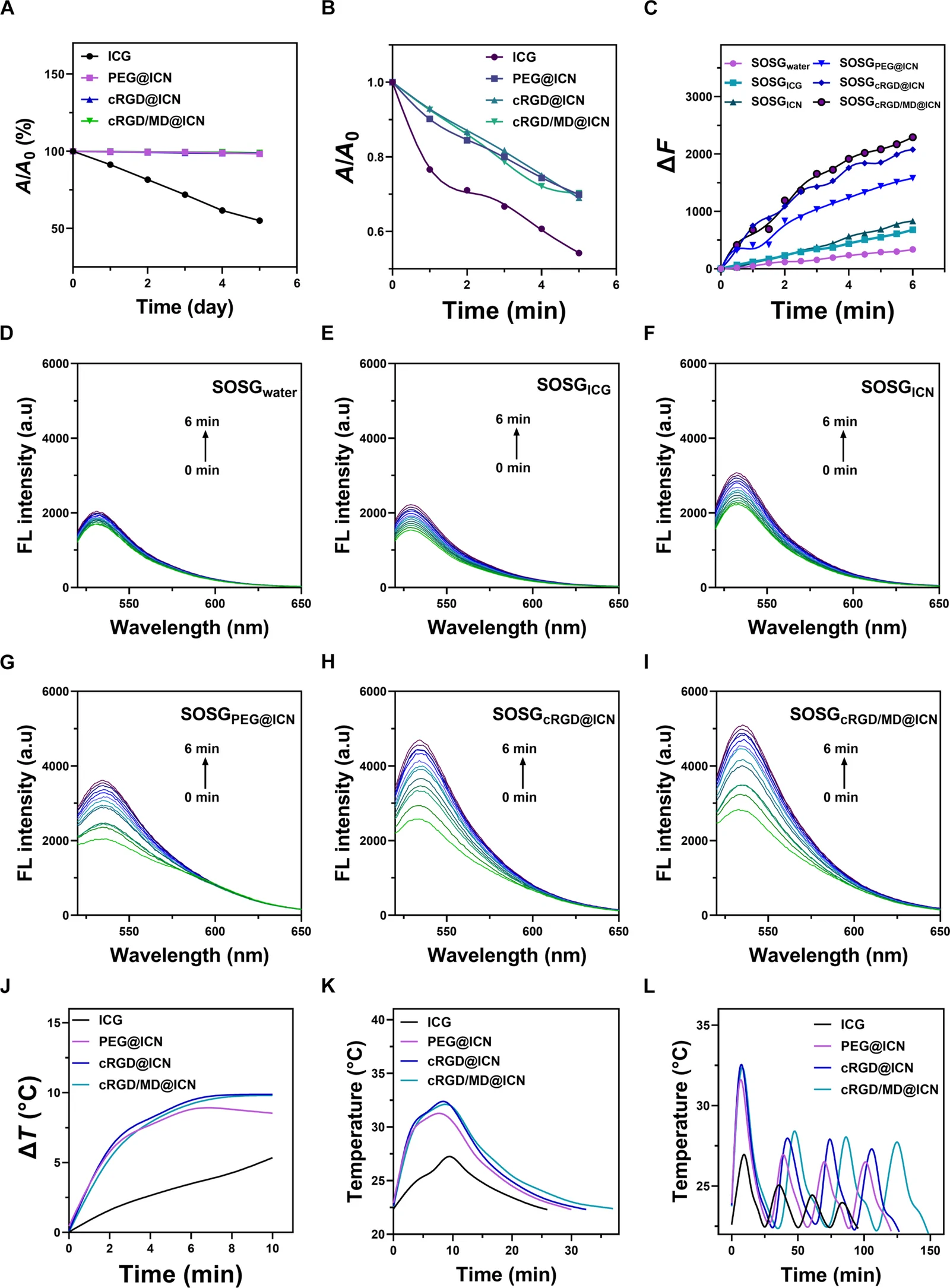
Photophysics features of different ICN formulations. (A) UV absorption changes of ICG at 784 nm in various samples after storage at 4 °C for 5 d. (B) Change of UV absorbance spectra of ICG samples (ICG concentration of 2.5 μg/ml, 808 nm, 0.5 W·cm^−2^, 5 min). (C) Fluorescence intensity changes of SOSG (5 μM) at 525 nm after incubation with different samples under irradiation (808 nm, 0.5 W·cm^−2^, 6 min). (D to I) Fluorescence intensity of SOSG (5 μM) in water and different ICG samples under 808-nm laser (0.5 W·cm^−2^) for 6 min (ICG concentration of 2.5 μg/ml). (J) Temperature changes of 5 μg/ml ICG samples upon exposure to 808-nm laser (0.5 W·cm^−2^) for different time. Temperature changes of ICG samples under laser irradiation for 1 (K) and 4 (L) cycles of heat-cooling experiment.

### Stability, photothermal response, and ^1^O_2_ generation ability of the nanoparticles

The stability of ICG in aqueous solution was assessed by UV–vis spectrophotometry. As shown in Fig. 3A, after 5 d, the absorbance of free ICG at 784 nm decreased by 45% but remained nearly unchanged for ICG encapsulated in nanoparticles. These results indicated that nanoencapsulation significantly enhanced ICG stability in aqueous solutions. To assess the photostability of cRGD/MD@ICN, free ICG and nanoparticles were irradiated with an 808-nm laser at a power density of 0.5 W·cm^−2^ for 5 min. The absorbance of free ICG decreased rapidly upon irradiation, whereas the nanoparticle formulation exhibited slower decrease of the absorbance (Fig. 3B and Fig. S4). These results further indicated that encapsulation also enhanced the photostability of ICG.

The in vitro photodynamic activity was assessed by monitoring temperature elevation and reactive oxygen species (ROS) generation under irradiation (808 nm, 0.5 W·cm^−2^, 10 min). In Fig. 3J to L, temperature increase for free ICG, PEG@ICN, cRGD@ICN, and cRGD/MD@ICN was 5.3, 8.5, 9.8, and 9.8 °C, respectively, demonstrating that the nanoparticles enhanced photothermal effects of free ICG. Moreover, cRGD/MD@ICN maintained stable photothermal performance over 4 cycles of consecutive laser, presenting the excellent photostablility in PTT.

The PDT efficacy of cRGD/MD@ICN was further evaluated using SOSG, a fluorescent indicator for singlet oxygen (^1^O_2_). As shown in Fig. 3D to I, the fluorescence intensity of SOSG at 525 nm (Fig. 3C) in nanoparticle solutions increased more progressively than free ICG. Notably, cRGD/MD@ICN showed the largest fluorescence enhancement.

### Cell uptake and anticancer toxicity assay of the nanoparticles

The cellular uptake of nanoparticles in 4T1 cells treated with various formulations was evaluated using confocal laser scanning microscopy (CLSM). As shown in Fig. 4A, red ICG fluorescence was barely observed in the free ICG group, while the fluorescence was brightest in cells treated with cRGD/MD@ICN. To verify the integrin receptor-mediated cell uptake, the cells were pretreated with DSPE-PEG-cRGD (1 mg/ml) for 2 h prior to cRGD/MD@ICN incubation. This competitive binding significantly reduced the internalization of cRGD/MD@ICN (Fig. 4A), as the free ligand DSPE-PEG-cRGD occupied the highly expressed α_ν_β_3_ integrin receptors on the surface of tumor cells [27,28]. We also found that pretreatment with MD peptides (1 mg/ml) inhibited the cell uptake of cRGD/MD@ICN, which suggested that the up-regulated MMP-2 in tumor may participate actively in regulating targeted internalization of cRGD/MD@ICN. Flow cytometry analysis quantitatively confirmed these findings (Fig. 4B and D and Fig. S5). Notably, the difference in uptake between cRGD/MD@ICN and free ICG became less obvious at higher concentrations (200 ng/ml for 2 h), probably due to a saturated situation of receptor binding under this condition. A time-dependent study further revealed enhanced accumulation of cRGD/MD@ICN in tumor cells over time (Fig. S5C and F), which would promote antitumor PDT in vivo.

**Fig. 4.**
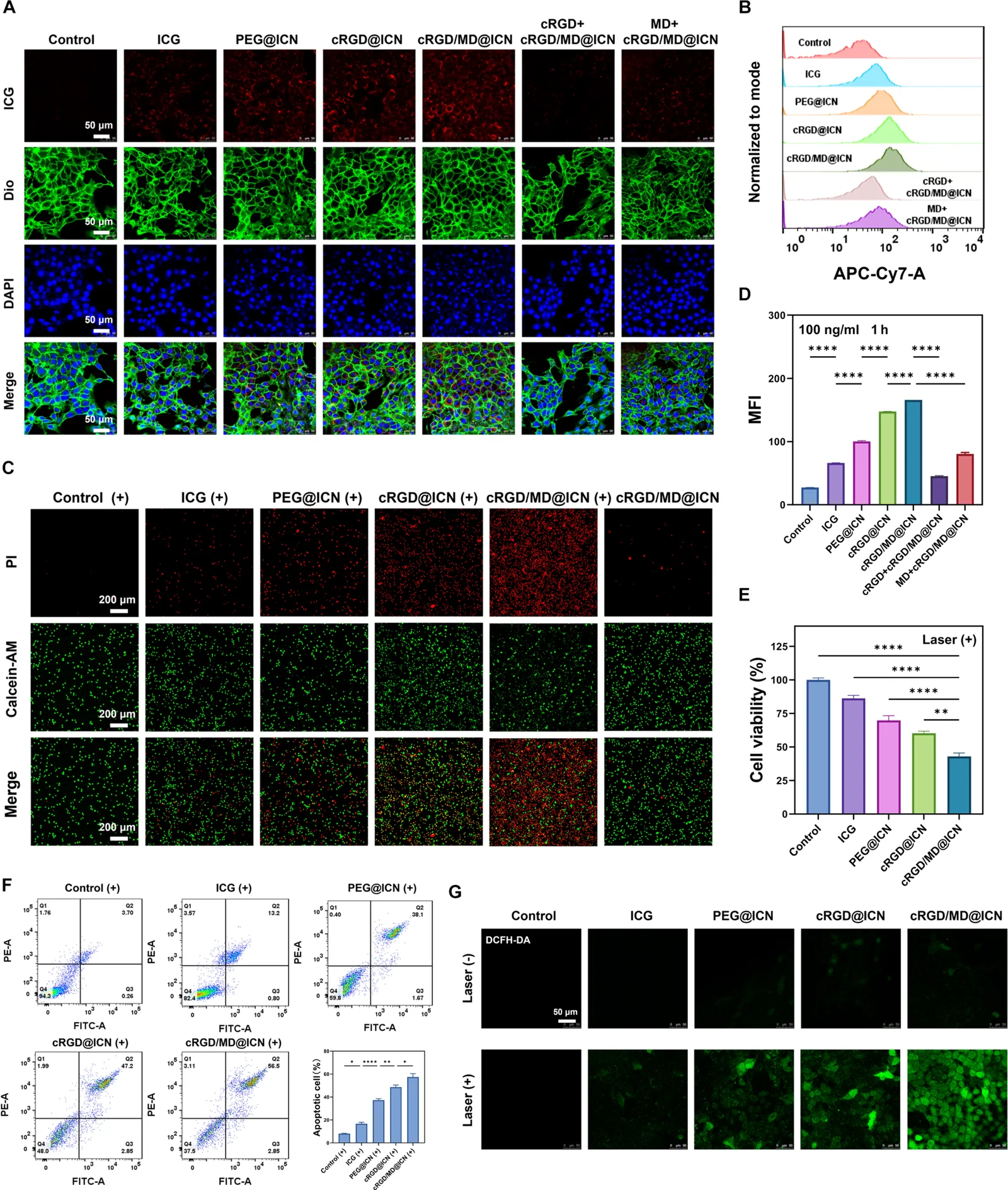
Cell uptake and phototherapy evaluation of the ICG nanoparticles. (A) CLSM images of 4T1 cells treated with different samples. (B and D) Intracellular ICG fluorescence intensity of 4T1 cells in different groups analyzed by flow cytometry (*n* = 3). (C) Calcein-AM and propidium iodide (PI) live-dead cell staining of 4T1 cells after treatment with different formulations with or without laser irradiation (808 nm, 0.5 W·cm^−2^, 5 min). (E) Cytotoxicity of the samples on 4T1 cells under 808-nm laser (0.5 W·cm^−2^) for 5 min (*n* = 3). (F) Flow cytometric quantification of Annexin V–fluorescein isothiocyanate (FITC)/PI-stained apoptotic 4T1 cells after exposure to different treatment groups (*n* = 3). (G) CLSM images of 4T1 cells after various treatments to track ROS by DCFH-DA (0.5 W·cm^−2^, 5 min). The ICG concentration is 3 μg/ml, and the notation “(+)” means laser irradiation. *P < 0.05, **P < 0.01, ****P < 0.0001.

The cytotoxicity of different nanoparticles toward 4T1 tumor cells was evaluated using the Cell Counting Kit-8 (CCK-8) assay. With no laser irradiation, cell viability remained above 90% following treatments (Fig. S6), indicating low cytotoxicity of the nanoparticles. Upon laser irradiation (808 nm, 0.5 W·cm^−2^, 5 min), cRGD/MD@ICN demonstrated the highest phototoxic effect (Fig. 4E). This photo-induced antitumor activity was further assessed using a dual live/dead staining assay. As shown in Fig. 4C, the cells in the cRGD/MD@ICN group exhibited more intense red fluorescence than the other groups after irradiation. Corresponding flow cytometry analysis (Fig. 4F) showed that the cRGD/MD@ICN + irradiation induced the highest apoptosis rate (56.5%), which was consistent with the results of cell viability and live/dead staining experiments.

### PDT effects and ICD responses of nanoparticles on 4T1 cells

Subsequently, intracellular ROS levels were monitored using the 2′,7′-dichlorodihydrofluorescein diacetate (DCFH-DA) fluorescent probe. After laser irradiation (808 nm, 0.5 W·cm^−2^, 5 min), all nanoparticle-treated cells showed green fluorescence, with the cRGD/MD@ICN group exhibiting the highest intensity (Fig. 4G and Fig. S7). As a crucial role in cancer immunotherapy, ICD can be triggered by multiple stimuli, including ROS and high level of intracellular calcium (Fig. 5B) [30,31]. The proposed mechanisms related to calcium overload-induced ICD are that a sudden increase in mitochondrial calcium uptake can induce the opening of the mitochondrial permeability transition pore (mPTP), leading to mitochondria dysfunction and even endoplasmic reticulum stress [23]. Together, these events promote the exposure and release of DAMPs, including CRT exposed to surface, HMGB1 secreted outside the cells, and ATP into the medium. These DAMPs serve as a potent “danger” signal to the immune system, ultimately eliciting a specific antitumor immune response. While PDT generates ROS that cause oxidative stress and subsequent ICD, similarly, PTT agents convert light into heat, leading to endoplasmic reticulum stress and ICD [32,33]. We first measured intracellular Ca^2+^ levels in 4T1 cells following incubation with the nanoparticle formulations. As shown in Fig. 5A, all nanoparticle-treated groups exhibited a significant increase in Ca^2+^ levels compared with the control group, with the cells in the cRGD/MD@ICN group showing the highest fluorescence intensity. Quantitative results of flow cytometry analysis further confirmed more amount of Ca^2+^ in cells of the cRGD/MD@ICN group (Fig. 5D and E).

**Fig. 5.**
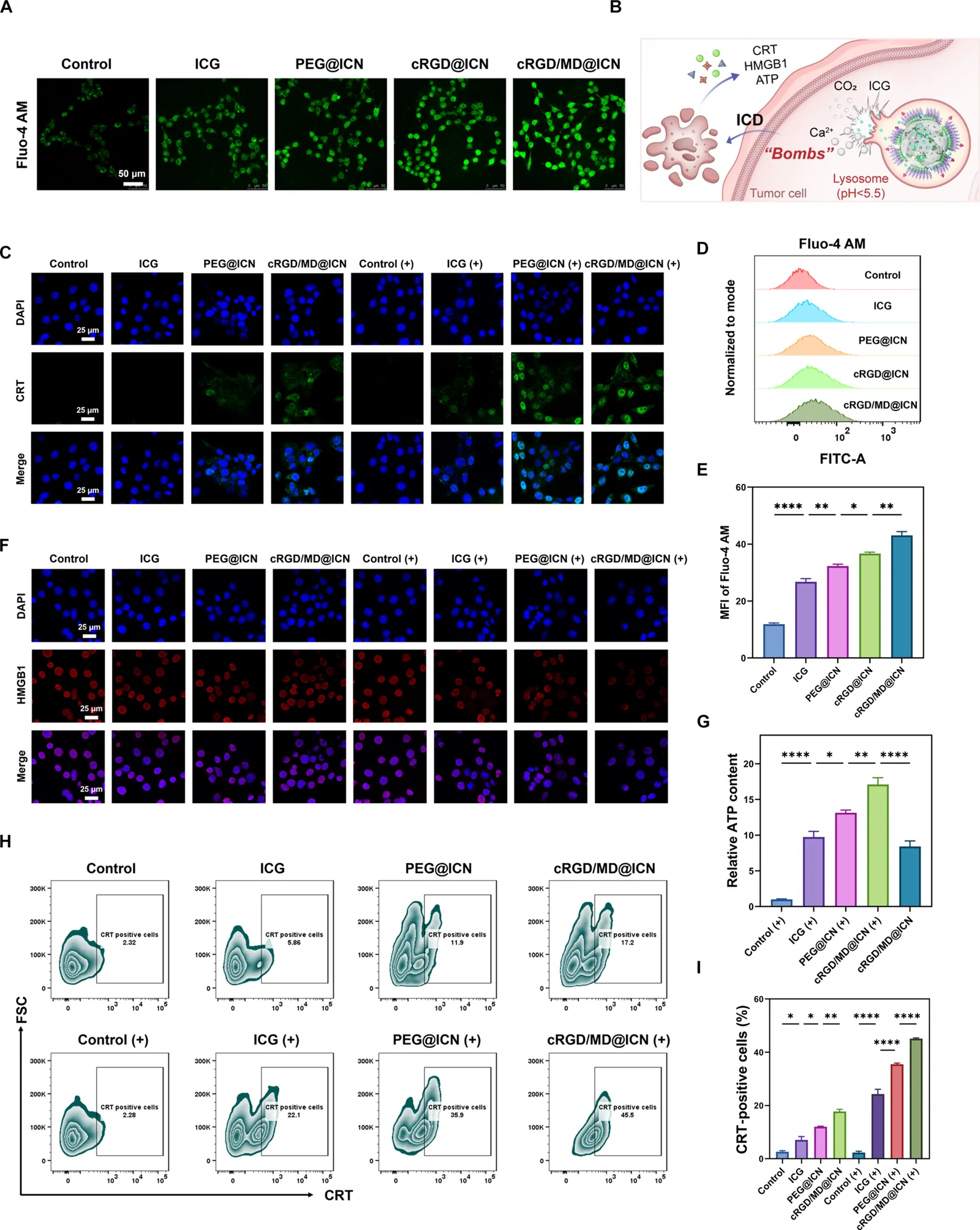
Intracellular Ca^2+^ production and ICD effect of ICG nanoparticles. (A) CLSM images and (D and E) flow cytometry analysis of Flou-4 AM to track Ca^2+^ production in cells after incubation with different samples. (B) Schematic diagram of calcium overload combined with ICG phototherapy to induce ICD. (C) Immunofluorescent staining of CRT and (F) HMGB1 from 4T1 cells treated with different groups. (G) ATP release by chemiluminescent assay (*n* = 4) and (H and I) flow cytometry analysis of CRT (*n* = 3) in 4T1 cells after different treatments. The ICG concentration is 3 μg/ml, and the notation “(+)” means laser irradiation. *P < 0.05, **P < 0.01, ****P < 0.0001.

To directly visualize lysosomal disruption, we performed LysoTracker colocalization assays in 4T1 cells treated with cRGD/MD@ICN. As shown in Fig. S5G, after incubation, the red fluorescence of cRGD/MD@ICN was largely separated from the green fluorescence of lysosomes, indicating successful lysosomal escape, consistent with the proposed “lysosome-bursting” mechanism. While this assay provides direct morphological evidence for lysosomal escape, a time-course study would better illustrate the progressive dynamics of lysosomal disruption. Nevertheless, the observed lysosomal escape, together with the consistent detection of Ca^2+^ release and subsequent ICD markers, supports the proposed mechanism.

Next, ICD was characterized in terms of CRT on the cell surface and the release of HMGB1. CRT expression in 4T1 cells treated with different formulations was examined by immunofluorescence and flow cytometry. Without laser irradiation, significant green fluorescence of CRT was observed in the cells of PEG@ICN and cRGD/MD@ICN groups than the free ICG group (Fig. 5C), indicating that the intracellular higher level of calcium can induce CRT exposure. Upon laser irradiation, the application of free ICG slightly induced CRT expression, while PEG@ICN and cRGD/MD@ICN treatment remarkedly enhanced green fluorescence, demonstrating the strong ability to promote CRT translocation (Fig. 5C). Flow cytometry results further confirmed these observations (Fig. 5H and I). The release of HMGB1 was also evaluated by immunofluorescence (Fig. 5F). In non-irradiated groups, PEG@ICN or cRGD/MD@ICN treatments caused slight reduction in nuclear red fluorescence of HMGB1, suggesting a little HMGB1 release. Under laser irradiation, the nuclear HMGB1 release was significantly promoted in the cells of ICG, PEG@ICN, and cRGD/MD@ICN groups, with the highest extent observed for the cRGD/MD@ICN + laser group (Fig. 5F). ATP release was measured simultaneously (Fig. 5G) and was 17-fold higher from the cells in the cRGD/MD@ICN + laser group than in the control group. Although ATP release can be triggered by multiple conditions, including the ICD process, conventional apoptosis, pyroptosis, and even necroptosis, the simultaneously promoted HMGB1 release and CRT translocation collectively demonstrated that cRGD/MD@ICN under laser irradiation can trigger a robustly high ICD response.

### In vivo biodistribution and antitumor phototherapy on orthotopic breast cancer

As combination actions of photothermal treatment and calcium overload synergistically induced more intense ICD effect, we then evaluated the tumor-targeting and accumulation properties of cRGD/MD@ICN before in vivo antitumor therapy. As shown in Fig. 6A and D, following intravenous administration, the fluorescence in tumor site of mice was significantly higher in the cRGD/MD@ICN group than in the free ICG group at all observed time points. In the cRGD/MD@ICN group, fluorescence intensity in tumors reached highest at 2 h post-injection and remained strong up to 8 h, while it rapidly declined over time for the free ICG group, indicating an enhanced retention of the nanoparticles in tumors. At 8 h post-injection, mice were euthanized, and tumors along with major organs were harvested for ex vivo imaging (Fig. 6B and E). For the free ICG group, fluorescence was predominantly localized in the liver and kidneys, with only weak signals detected in tumor tissue. In contrast, both PEG@ICN and cRGD/MD@ICN highly accumulated in tumors, and cRGD/MD@ICN exhibited the highest tumor-specific fluorescence, further confirming its superior tumor-targeting and enrichment capability.

**Fig. 6.**
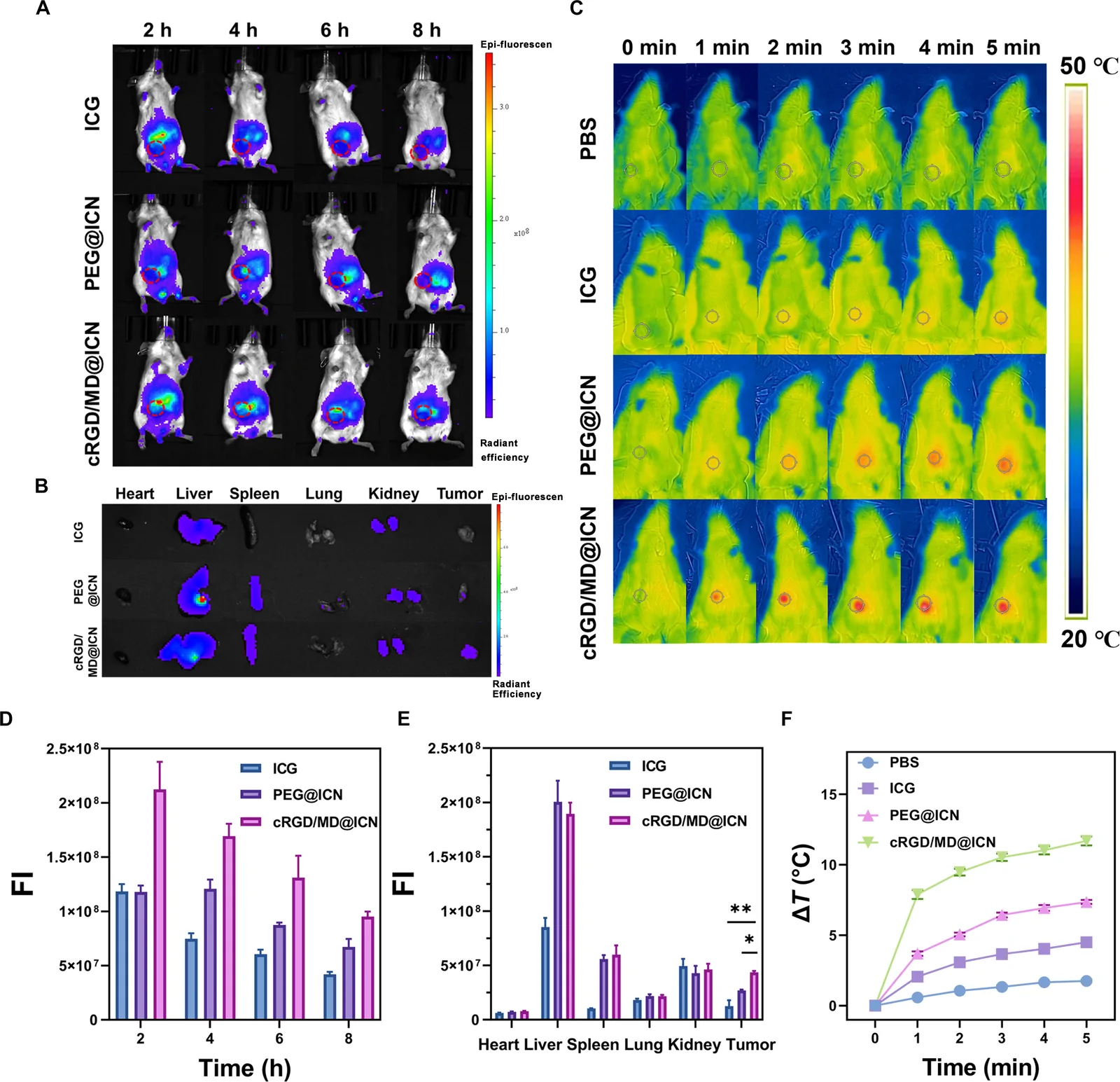
In vivo imaging and photothermal responses of ICG formulations in mice. (A) In vivo fluorescence imaging and (D) intensity of ICG formulations in 4T1 tumor-bearing mice at different time points after intravenous injection (100 μl, *n* = 3). (B) Fluorescence micrographs and (E) signal quantification of major organs isolated from 4T1-bearing mice 8 h after treatment with different nanoparticle formulations (excitation = 710 nm; emission = 800 nm) (*n* = 3). (C) Thermal images and (F) tumor temperatures of 4T1 tumor-bearing mice in different groups treated under NIR laser irradiation (808 nm, 0.5 W·cm^−2^, 5 min) (ICG concentration of 200 μg/ml). *P < 0.05, **P < 0.01.

As for PTT-based immunotherapy, high-temperature PTT can rapidly disrupt tumor cell membranes and induce cell necrosis, but may also damage surrounding normal tissues, leading to vascular injury, and even potential immunosuppression [34]. In contrast, mild photothermal treatment around 41 to 45 °C can greatly minimize damage to normal tissues, but maintain the higher ICD effect [35,36]. Therefore, a lower laser intensity (808 nm, 0.5 W·cm^−2^, 5 min) was performed for in vivo antitumor treatment. In Fig. 6C and F, tumor temperature in the cRGD/MD@ICN group increased by approximately 11.7 °C (to about 45 °C) within 5 min of irradiation, while only temperature increase of 4.5 °C was observed in the free ICG group, showing a superior photothermal performance of cRGD/MD@ICN at the tumor site.

An orthotopic 4T1 breast cancer model was established in BALB/c mice to evaluate the in vivo antitumor efficacy of cRGD/MD@ICN (Fig. 7A). 4T1 cells, a well-established and widely acceptable in vivo tumor model of triple-negative breast cancer (TNBC), have been widely used in the studies of both primary and metastatic tumors [3]. Mice were intravenously administered 100 μl of phosphate-buffered saline (PBS), ICG, PEG@ICN, or cRGD/MD@ICN (ICG concentration: 200 μg/ml). Four hours post-injection, tumors were irradiated with an 808-nm laser (0.5 W·cm^−2^, 5 min). As seen in Fig. 7B, tumor growth in the mice of PBS, PBS + laser, ICG, and PEG@ICN groups was similar, indicating that either laser irradiation alone or nanoparticle administration with no laser did not significantly inhibit the tumor growth. The treatments of ICG + laser and PEG@ICN + laser showed modest tumor inhibition. In contrast, the cRGD/MD@ICN group demonstrated superior tumor suppression. This efficacy may be the synergistic effect of multiple actions: (a) cRGD-mediated active targeting promoted nanoparticle accumulation in tumor tissue; (b) the high expression of MMP-2 in the tumor microenvironment triggered the release of the functional peptide ^D^PPA-1, which competitively blocked the PD-L1/PD-1 immune checkpoint, thereby alleviating immunosuppression; and (c) calcium overload and localized photothermal action under laser irradiation directly induced tumor cell damage and ICD effects. Together, these effects resulted in potent inhibition of orthotopic 4T1 tumor growth. Pictures of tumors excised from the mice further confirmed that cRGD/MD@ICN + laser demonstrated the optimal antitumor efficacy (Fig. 7D). Hematoxylin and eosin (H&E) staining of tumor sections revealed substantial cellular damage and nuclear deformation in the cRGD/MD@ICN plus laser group. Terminal deoxynucleotidyl transferase-mediated deoxyuridine triphosphate nick end labeling (TUNEL) staining results indicated that this treatment induced significantly higher levels of apoptosis/necrosis compared to other groups. Additionally, Ki67 expression in tumors from the cRGD/MD@ICN + laser group was markedly reduced, confirming the suppression of tumor cell proliferation. In summary, the histopathological and immunohistochemical results demonstrated the strong therapeutic potential of cRGD/MD@ICN in antitumor treatment. Throughout the treatment period, mouse body weight remained stable (Fig. 7C), and H&E staining of major organs revealed no obvious pathological changes (Fig. S8). Additionally, plasma biochemical parameters [alanine aminotransferase (ALT), aspartate aminotransferase ( AST), creatinine (CRE), uric acid (UA), and blood urea nitrogen (BUN)] in mice of nanoparticle groups were statistically comparable with the PBS group (Fig. S9), suggesting favorable biocompatibility and low systemic toxicity of the nanotreatment.

**Fig. 7.**
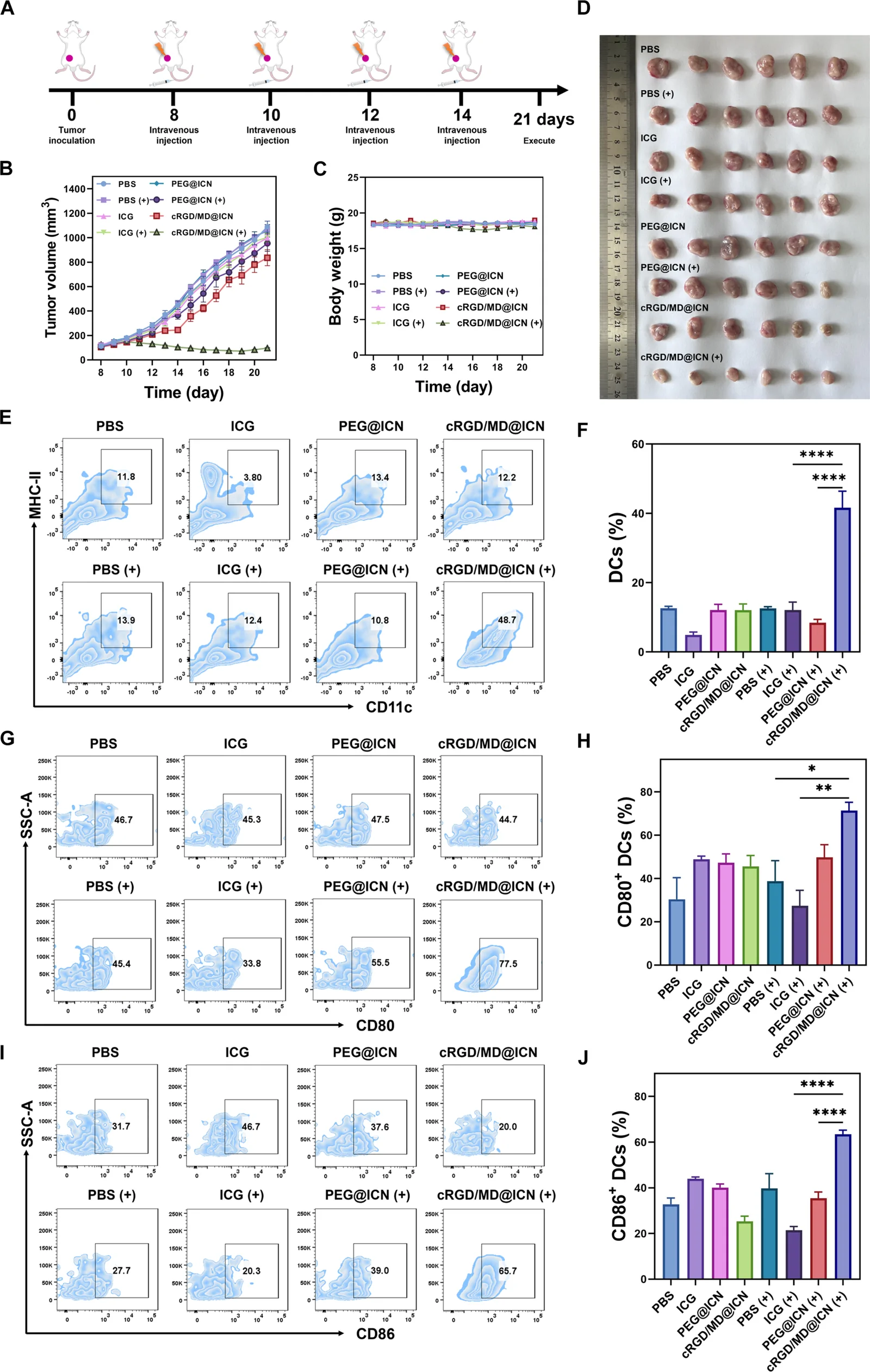
In vivo antitumor phototherapy and matured DC analysis in BALB/c mice. (A) Treatment scheme of the antitumor studies. (B) Average tumor volume and body weight (C) of the mice with different treatments (*n* = 4 to 6). (D) Photographs of tumors in different groups at the end of antitumor treatments. Flow cytometry analysis of (E and F) matured DC, (G and H) CD80^+^, and (I and J) CD86^+^ mature DC in tumors of each group (*n* = 4). The notation “(+)” means laser irradiation. *P < 0.05, **P < 0.01, ****P < 0.0001.

After the treatment was finished, flow cytometry analysis of tumor-associated DCs showed that the cRGD/MD@ICN plus laser group stimulated DC maturation rate to 41.6%, with 71.4% and 63.5% of matured DCs expressing CD80^+^ and CD86^+^, respectively (Fig. 7E to J and Fig. S10). We further evaluated immune cell proportion in the spleen and found that the cRGD/MD@ICN plus laser treatment induced the highest proportion of CD3^+^ T cells (21.15%), among which CD8^+^ cytotoxic T cells accounted for 33.67% (Fig. 8 and Fig. S11).

**Fig. 8.**
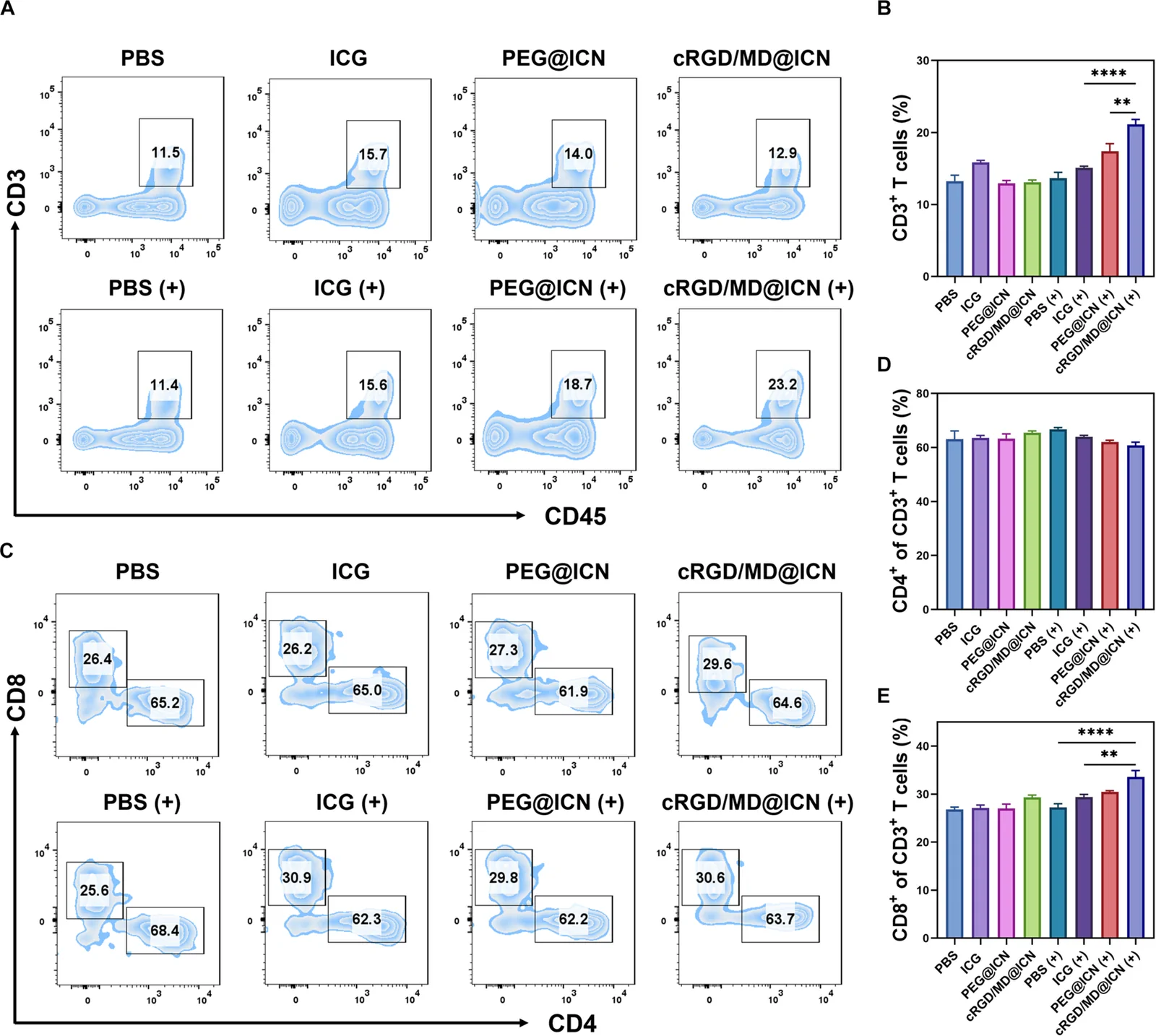
In vivo antitumor phototherapy analysis of T cells in BALB/c mice. (A and B) Flow cytometry analysis of CD3^+^ T cells in spleens (*n* = 4). (C to E) Flow cytometry analysis of CD4^+^ T cells and CD8^+^ T cells in spleens among CD3^+^ T cells (*n* = 4). The notation “(+)” means laser irradiation. **P < 0.01, ****P < 0.0001.

The immunogenic mechanism was then validated by immunofluorescence analysis of tumor sections. As seen in Fig. 9, the exposure of CRT and release of HMGB1 was observed in tumor, indicating the inducing of ICD by the phototherapy. During the ICD process, the released DAMPs promote DC maturation, which would then initiate antigen-specific antitumor immune responses. These results demonstrated that cRGD/MD@ICN-based photoimmunotherapy effectively promoted DC maturation and antigen-presenting capability, and then activated CD8^+^ T cell to facilitate tumor-specific immune responses. Immunofluorescence staining revealed strong infiltration of CD4^+^ T and CD8^+^ T cells in tumor following phototherapy (Fig. 9). Consistent with findings in Fig. 7E to J, immunofluorescence staining showed highest proportions of CD11c^+^/CD80^+^ and CD11c^+^/CD86^+^ cells in tumors after laser treatment with cRGD/MD@ICN (Fig. 9). Although direct in vivo visualization of calcium overload remains technically challenging, the consistent detection of ICD-associated markers in tumor tissues, together with the observed DC maturation and T cell infiltration, provides indirect evidence that the proposed lysosome-bursting and calcium overload mechanisms are operative in vivo. Future studies employing genetically encoded calcium indicators or intravital imaging approaches may further consolidate this mechanistic understanding. In summary, cRGD/MD@ICN modulated the tumor immune microenvironment through PD-L1 blockade and synergistic phototherapy, thereby promoting DC maturation and cytotoxic T cell activation. Considering that tumor immune response is a complex process mediated by diverse immune cell populations, in future study, we will expand immune monitoring toward additional immune subsets, such as myeloid-derived suppressor cells (MDSCs) and regulatory T cells (Tregs), to enable a more comprehensive understanding of the tumor immune microenvironment.

**Fig. 9.**
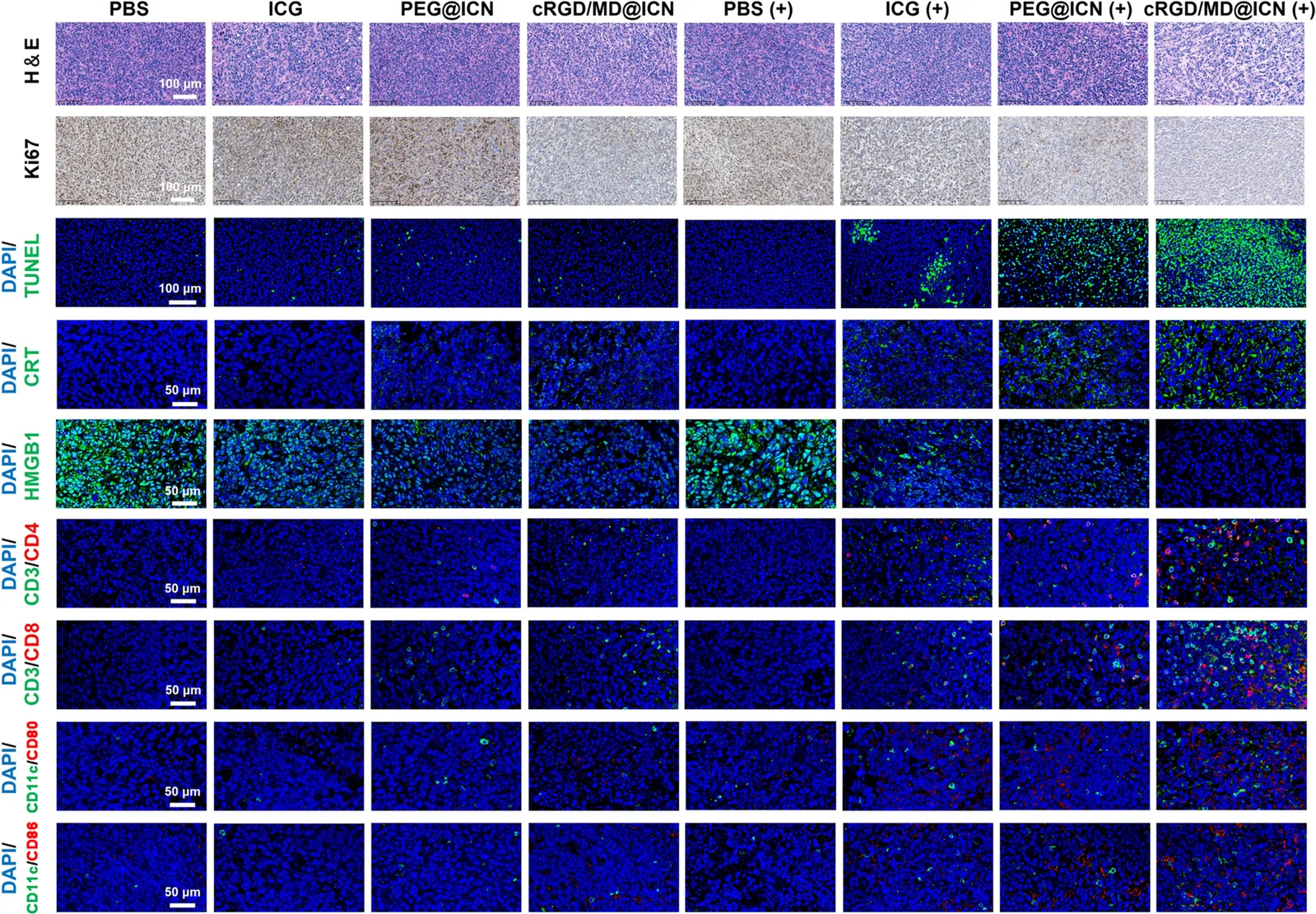
H&E staining, immunohistochemistry staining of Ki67, and immunofluorescence staining of TUNEL, CRT, HMGB1, CD3/CD4, CD3/CD8, CD11c/CD80, and CD11c/CD86 of the tumors after the in vivo treatments. The notation “(+)” means laser irradiation.

### Antitumor immunotherapy on pulmonary metastasis from breast cancer

To evaluate the antimetastatic efficacy of cRGD/MD@ICN-based photoimmunotherapy, we established lung metastatic of breast cancer by inoculation of orthotopic 4T1 breast cancer cells, followed by intravenous injection of 4T1-luc cells to track metastasis (Fig. 10A and B). Treatment protocols were similar with antitumor study above, and the metastatic progression was monitored by bioluminescence imaging on days 13, 18, and 21 using a small animal imaging system. Antitumor effects on the orthotopic tumor were consistent with in vivo antitumor studies described above (Fig. 10C and Fig. S12). During treatment, body weight was basically the same among groups (Fig. 10D). Bioluminescence imaging results (Fig. 10E and G) revealed that lung fluorescence appeared in the groups of PBS + laser, ICG + laser, and cRGD/MD@ICN without laser on day 18, indicating the occurrence of lung metastasis of breast cancer. By day 21, much brighter fluorescence was observed in nearly all mice in these groups. Among these therapies, cRGD/MD@ICN + laser treatment displayed the strongest suppression effect on the lung metastasis, with the lowest fluorescence in pulmonary and primary tumors. Direct examination of excised lungs showed fewer visible nodules in mice of the cRGD/MD@ICN + laser group compared with other groups (Fig. 10H). Quantification analysis confirmed an average of only 7 nodules in the cRGD/MD@ICN + laser group, decreased by approximately 91% relative to the PBS group (Fig. 10F). H&E staining of lung sections (Fig. 10I) further proved no metastatic lesions in the cRGD/MD@ICN + laser group.

**Fig. 10.**
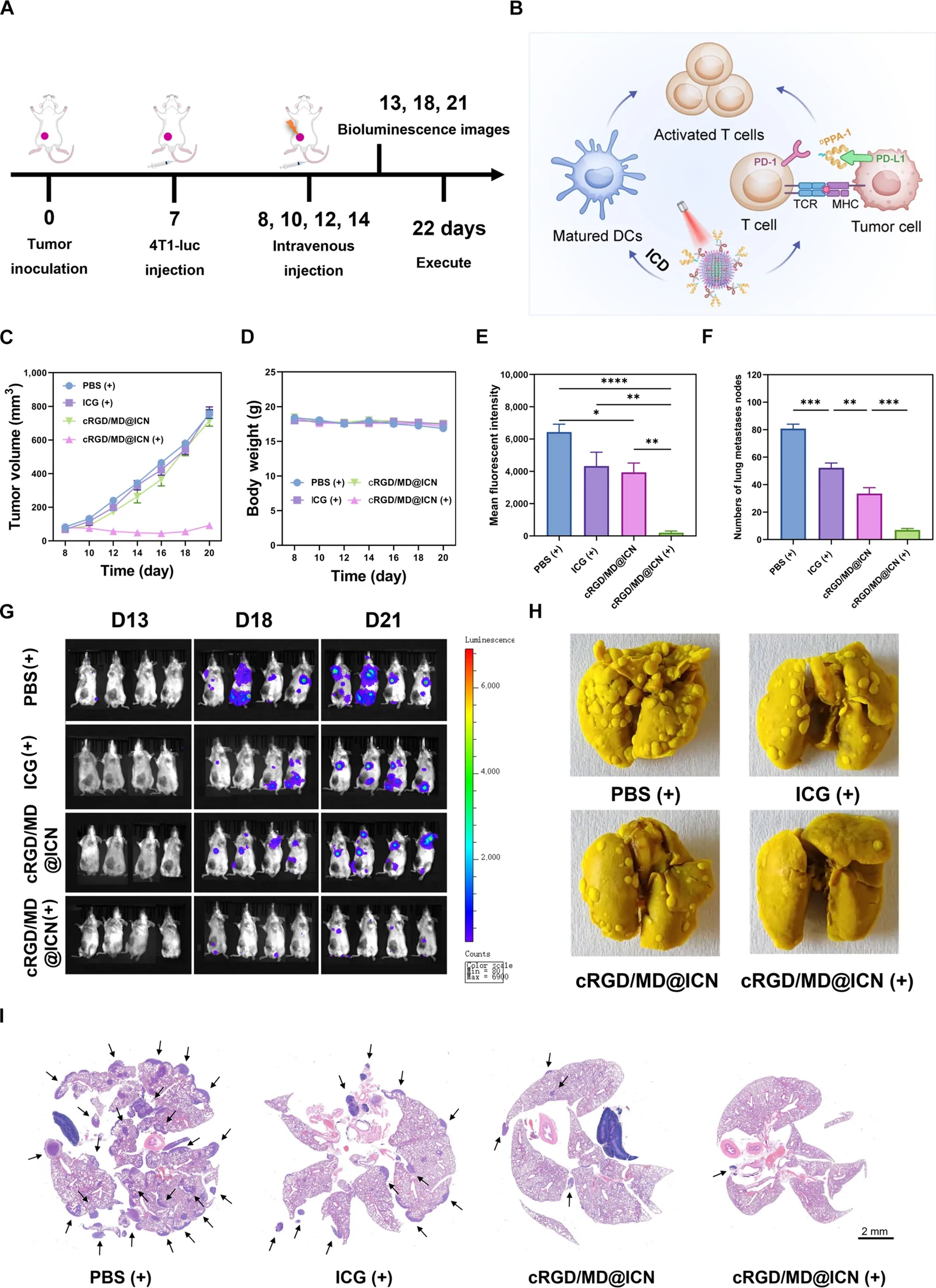
Antitumor immunotherapy against lung metastasis of breast cancer in BALB/c mice. (A) Schematic illustration of animal model development and treatments. (B) Proposed mechanism of cRGD/MD@ICN phototherapy in the antitumor immunotherapy. (C) Average tumor volume and body weight (D) of the mice with different treatments (*n* = 4). (G) In vivo bioluminescence images and (E) intensity of the 4T1-luc cells to track the growth of the tumor cells in different treatment groups (*n* = 4). (F) Numbers and (H) photographs of lung nodules in different groups at the end of treatments (*n* = 4). (I) H&E staining of the lung metastasis, with the black arrows indicating the nodules. The notation “(+)” means laser irradiation. *P < 0.05, **P < 0.01, ***P < 0.001, ****P < 0.0001.

When the therapy was completed, we quantified T cell subsets in mouse tumor tissues by flow cytometry analysis (Fig. 11). The cRGD/MD@ICN + laser treatment induced the largest infiltration of CD3^+^ T cells into tumor (14.4%), indicating robust immune recruitment. Although the differences in CD8^+^CD69^+^ and CD4^+^CD69^+^ subsets did not reach statistical significance, the overall infiltration of CD3^+^ T cells and the ratio of CD8^+^ to CD4^+^ T cells were significantly elevated in the cRGD/MD@ICN plus laser group. These observations, together with the reduced lung metastatic nodules, support enhanced T cell-mediated antitumor immunity. Future studies with additional time points will be needed to clarify the activation kinetics of CD69^+^ subsets. These immunophenotypic changes demonstrated that the combined therapy greatly inhibited metastasis via enhanced T cell-mediated immunity.

**Fig. 11.**
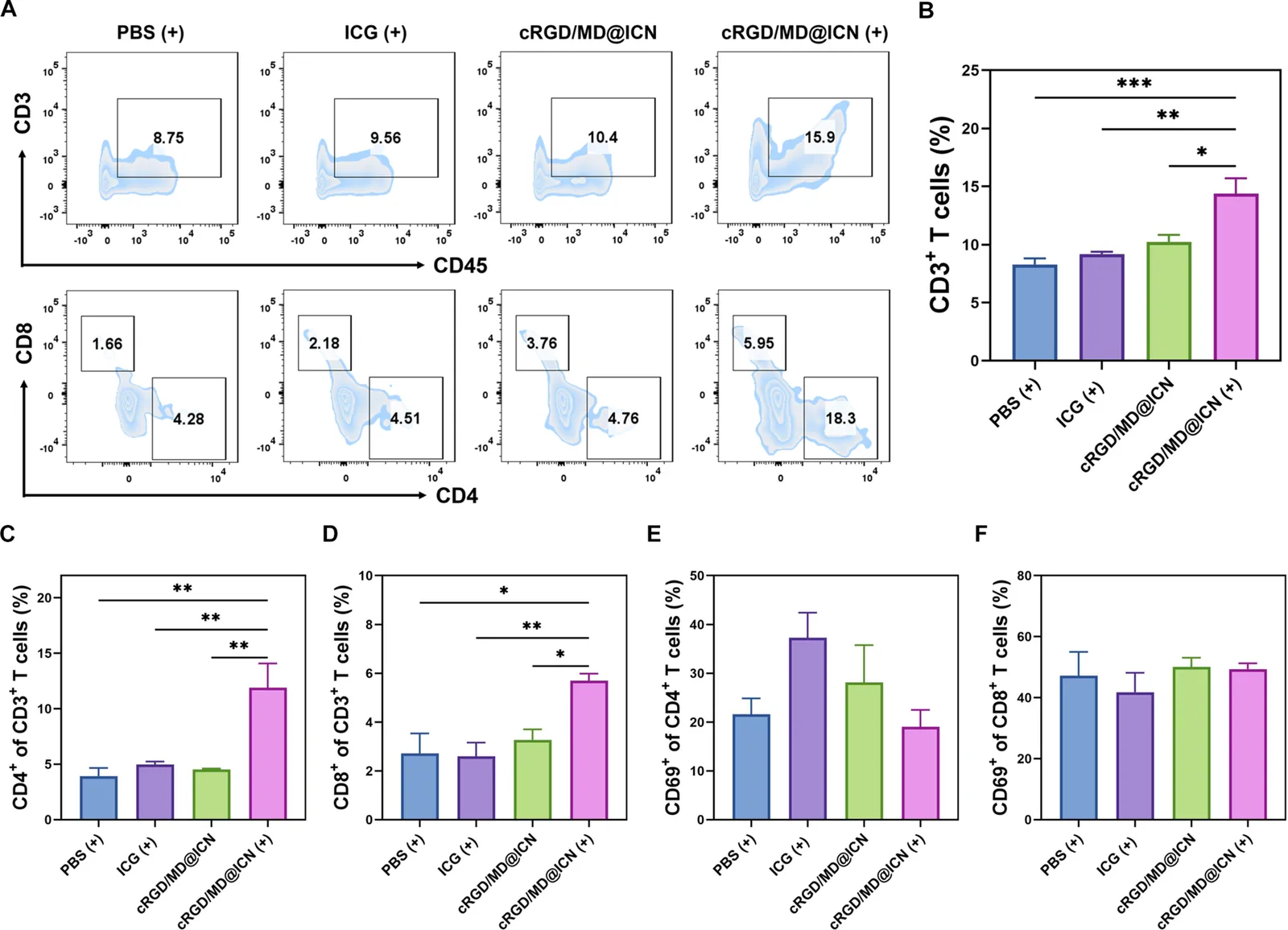
Evaluation of in vivo immune responses induced by phototherapy against tumor metastasis. (A and B) Flow cytometry analysis of CD3^+^ T cells in tumors (*n* = 4). (C and D) Flow cytometry analysis of CD3^+^CD4^+^ and CD3^+^CD8^+^ T cells in tumors (*n* = 4). (E and F) Flow cytometry analysis of CD4^+^CD69^+^ T cells and CD8^+^CD69^+^ T cells in tumors (*n* = 4). The notation “(+)” means laser irradiation. *P < 0.05, **P < 0.01, ***P < 0.001.

## Conclusion

In this study, we successfully developed a lysosome-bursting calcium carbonate nanoplatform (cRGD/MD@ICN) that integrates phototherapy, calcium overload, and ICB to amplify ICD for enhanced cancer immunotherapy. The nanosystem leverages the pH-responsive dissolution of calcium carbonate within tumor lysosomes to trigger rapid Ca^2+^ release and lysosomal rupture, thereby inducing calcium overload-mediated ICD. Concurrently, the encapsulated ICG exerts potent photodynamic and photothermal effects under NIR irradiation, synergizing with calcium overload to achieve dual amplification of ICD, as evidenced by enhanced CRT exposure, HMGB1 release, and ATP secretion. The MMP-2-responsive exposure of ^D^PPA-1 effectively blocks the PD-1/PD-L1 pathway, further relieving immunosuppression in the tumor microenvironment. With cRGD-mediated active tumor targeting, cRGD/MD@ICN demonstrates superior tumor accumulation and retention, leading to robust inhibition of both orthotopic breast tumor growth and pulmonary metastasis in vivo. This work presents a promising strategy that harnesses lysosome-bursting calcium carbonate nanoparticles to synergize phototherapy and calcium overload for ICD amplification, offering extended applications in ICB and advance cancer photo-immunotherapy.

## Materials and Methods

Detailed information regarding all materials and methods is available in the Supplementary Materials.

## Data Availability

The data supporting the results of this study can be obtained from the corresponding author upon reasonable request.
