# Supplementary material for: Lysosome-Bursting Calcium Carbonate Nanoparticles Amplify Immunogenic Cell Death via Synergistic Phototherapy and Calcium Overload for Immune Checkpoint Blockade
Source: Research (Wash D C). 2026 Oct 12;9:1444. doi: 10.34133/research.1444 (PMC13655353; doi:10.34133/research.1444)
Supplement: Supplementary file 1 [file research.1444.f1.docx]

**Supplementary Materials**

**Lysosome-Bursting Calcium Carbonate Nanoparticles Amplify Immunogenic Cell Death via Synergistic Phototherapy and Calcium Overload for Immune Checkpoint Blockade**

**2. Methods and Materials**

**2.1 Materials**

Indocyanine green (ICG) was purchased from Bide Pharmatech Co., Ltd (Shanghai, China). Calcium chloride dihydrate (CaCl_2_·2H_2_O) was purchased from Shanghai Yuanye Bio-Technology Co., Ltd (Shanghai, China). Ammonium hydrogen carbonate (NH_4_HCO_3_) was purchased from Tianjin Bohua Chemical Reagents Co., Ltd (Tianjin, China). Ethanol was purchased from Jiangtian Chemical Technology Co., Ltd (Tianjin, China). Soybean lecithin (Lipoid S100) was obtained from AVT Pharmaceutical Tech Co., Ltd (Shanghai, China). Ponsure (Shanghai, China) was the supplier of DSPE-PEG2000 and DSPE-PEG2000-Mal. DSPE-PEG2000-cRGD was obtained from Ruixi Biotech Co., Ltd (Xian, China), and the MMP-2-^D^PPA-1 (MD) peptide (sequence: CPLGLAGNYSKPTDROYHF) was acquired from GL Biochem Ltd (Shanghai, China). Gibco supplied the RPMI 1640 medium. Sino Biological (Beijing, China) provided the fetal bovine serum (FBS). LABLEAD (Beijing, China) provided the Annexin V/PI apoptosis kit, and the Fluo-4 AM (F8501) and ATP assay kits (BC0300) were purchased from Solarbio (Beijing, China). The antibodies utilized for flow cytometry were obtained from BioLegend (San Diego, USA).

**2.2 Cells and animals**

The 4T1 and 4T1-luc cell lines were sourced from the Cell Culture Center at the Institute of Basic Medical Sciences, Peking Union Medical College and from Shanghai Sciencelight Biology Science & Technology, respectively. All cells were cultured at 37°C in 5% CO_2_. Female BALB/c mice (6 weeks, 17–20 g) were provided by Beijing Vital River Laboratory Animal Technology Co. Ltd.

**2.3 Preparation of different types of ICG nanoformulations**

Firstly, we prepared calcium carbonate nanoparticles encapsulating ICG (ICN) via gas diffusion method. First, 200 mg of CaCl_2_•2H_2_O and 1 mg of ICG (pre-dissolved in water) were dissolved in 100 mL of anhydrous ethanol. The solution was then placed in a conical flask covered with perforated aluminum foil. This flask, together with another flask containing an appropriate amount of NH_4_HCO_3_ powder, was placed in a sealed container, and maintained at 25°C for 48 h. Then, the product was centrifuged at 10,000 rpm to obtain the final ICN product in ethanol which was stored at 4°C for subsequent use.

For ICN modification, DSPE-PEG2000-MD was synthesized by covalently conjugating DSPE-PEG2000-maleimide (DSPE-PEG2000-Mal) with the MD peptide (sequence: CPLGLAGNYSKPTDROYHF) at a molar ratio of 1.2:1 (peptide to polymer). The reaction was conducted in DMF under continuous stirring at 25°C for 24 h. The crude product was then purified through dialysis against deionized water for a period of 24 h, employing a dialysis bag with a 4 kDa molecular weight cut-off. The DSPE-PEG2000-MD conjugate was ultimately freeze-dried and kept at -20°C for later use. Its chemical structure was analyzed using ^1^HNMR and MALDI-TOF.

After that, various kinds of ICN liposomes were prepared by the thin-film dispersion method, including PEG@ICN, cRGD@ICN, and cRGD/MD@ICN. Specially, S100 phospholipids and ICN (1:1, w/w) were dissolved in ethanol-chloroform mixture (1:1, v/v). After ultrasonication and stirring at 37°C for 7 h, the solvent was rotary-evaporated at 45°C to form a dry film. The film was dissolved in anhydrous ethanol and mixed with the PEGylated lipids according to the following formulations: DSPE-PEG-cRGD: DSPE-mPEG2000: S100 = 7:100:535 (weight ratio) for cRGD@ICN; DSPE-PEG-cRGD: DSPE-mPEG2000: DSPE-PEG-MD: S100 = 8:55:100:815 (weight ratio) for cRGD/MD@ICN. The lipid solutions were then sonicated (100 W, 10 min, pulsed for 3 s between each 3 s) and injected into 18 mL of water under stirring. The final products were obtained after removing residual ethanol by rotary evaporation at 37°C.

**2.4 Characterization and pH-responsive release of the nanoparticles**

The sizes and zeta potentials of ICN, PEG@ICN, cRGD@ICN, and cRGD/MD@ICN were determined using a Zeta Sizer from Malvern. The nanoparticles' morphology was analyzed with cryo-TEM (FEI Talos, USA). The elemental mapping images were analyzed by transmission electron microscope (TEM, JEM-2800, Japan).

To detect pH-responsive release calcium ion from the nanoparticles, cRGD/MD@ICN (2 mL) was dispersed in 20 mL of PBS at different pH values (5.5, 6.5, and 7.4) and loaded into dialysis bags (MWCO = 4000), then shaken at 100 rpm and 37°C. At designed time points, 250 μL of the release medium was taken and immediately replaced with an equal volume of fresh PBS. After centrifugation of the collected samples at 10,000 rpm and 4°C for 10 min, the supernatant was analyzed for Ca^2+^ concentration using a Calcium Assay Kit.

**2.5 Photothermal response and** **^1^O_2_ generation ability** **of nanoparticles**

The photothermal effect was evaluated by exposing ICG solution and nanoparticle samples with 5 μg/mL ICG to an 808 nm NIR laser at a power density of 0.5 W·cm^-2^. For ^1^O_2_ detection, the detected sample solutions were mixed with the SOSG probe at a final concentration of 5 μM. Following laser irradiation (808 nm, 0.5 W·cm^-2^) for 6 min, the fluorescence emission of SOSG at 525 nm was measured at 30-second intervals to quantify ^1^O_2_ generation.

**2.6 Cell uptake and anticancer toxicity assay of the nanoparticles**

Cellular internalization was visually imaged by laser scanning confocal microscope. The 4T1 cells were counted using a Countstar automated cell counter (Countstar Mira FL, Shanghai, China) and cultured for 24 h after being seeded in confocal dishes. The cells were then incubated with different nanoparticles for an additional 24 h. Following incubation, the cells were washed, fixed, and then stained with DiO for cell membrane and DAPI for nuclei imaging. Cellular uptake was visualized using a Leica laser scanning confocal microscope (TCS SP8, Leica, Germany) (Ex = 633 nm, Em = 650 - 800 nm for nanoparticle groups). To further investigate the targeting mechanism, a competitive inhibition cell uptake was tested. Cells were pretreated for 2 h with either 1 mg/mL DSPE-PEG-cRGD or MD peptide before addition of cRGD/MD@ICN. For quantitative analysis, after the same treatments, cells were analyzed using a flow cytometer (BD LSRFortessa).

For assessment of anticancer effect, 4T1 cells were plated and cultured for 24 h. The cells were then treated with ICG, PEG@ICN, cRGD@ICN, or cRGD/MD@ICN for 24 h, and exposed to NIR laser irradiation during this period. Following the treatments, cell viability was assessed. The percentage of cell viability was determined using the equation: Cell viability (%) = [(As - Ab) / (Ac - Ab)] × 100%, where As, Ac and Ab are absorbances of treated cells, untreated cells and blank control, respectively.

Live/dead staining and flow cytometry were also performed in 4T1 cells. Cells were plated in a 24-well plate overnight and then treated with different ICG formulations (3 μg/mL) for 12 h, receiving NIR laser irradiation (808 nm, 0.5 W·cm^-2^, 5 min). For live/dead staining, the treated cells were harvested, stained with Calcein-AM and PI for 15 min, and visualized by fluorescence microscopy. For apoptosis analysis, the treated cells were subjected to Annexin V-FITC/PI staining for 15 min, and analyzed by flow cytometry, with data processed using FlowJo software (BD LSRFortessa).

**2.7 PDT effects and ICD responses of nanoparticles on 4T1 cells**

4T1 cells were plated in confocal dishes overnight and subsequently incubated for 24 h with 3 μg/mL ICG or nanoparticle formulations. Prior to imaging, cells were incubated with DCFH-DA and irradiated with an NIR laser (808 nm, 0.5 W·cm^-2^, 5 min). Fluorescence images were then acquired immediately using a Leica fluorescence microscope to indicate ROS levels.

Intracellular retention of Ca^2+^ in 4T1 cells were detected using the Fluo-4 AM probe. Cells were treated with 3 μg/mL ICG formulations for 24 h. The treated cells were then incubated with 2 μM Fluo-4 AM for 30 min at 37°C. For qualitative analysis, the Fluo-4 AM fluorescence was immediately imaged by confocal microscopy. For quantitative analysis, the treated cells were harvested, and fluorescence intensity was measured using a flow cytometer.

The expression of CRT and release of HMGB1 protein were assessed by immunofluorescence staining. 4T1 cells were cultured overnight, then co-incubated with different ICG nanoparticle formulations (3 μg/mL) for 12 h, after which the phototherapy groups were exposed to NIR laser irradiation (808 nm, 0.5 W·cm^-2^, 5 min). Following treatments, after fixation with 4% paraformaldehyde for 15 min, the cells were permeabilized with 0.5% Triton X-100 for an additional 20 min. Non-specific binding sites were blocked with 5% BSA for 45 min. The cells were then incubated overnight at 4°C with primary antibodies, including Alexa Fluor 488-conjugated anti-CRT antibody and unlabeled anti-HMGB1 antibody. After washing, the samples were incubated with Alexa Fluor 647-labeled secondary antibody to bind HMGB1 primary antibody for 2 h at room temperature. Nuclei were stained with DAPI. After final washes with TBST buffer, the samples were imaged using CLSM (TCS SP8, Leica, Germany).

For flow cytometric analysis of CRT expression, cells were harvested by trypsinization and stained with an Alexa Fluor 488-conjugated anti-CRT antibody for 1 h prior to analysis. To assess ATP release, cells were treated as described above, and the resulting supernatants were collected for detection using a chemiluminescence-based assay kit.

**2.8 *In vivo* biodistribution and antitumor phototherapy**

Female BALB/c mice (6 weeks old) were obtained from Vital River Laboratory Animal Technology Co., Ltd. All animal experiments were conducted in compliance with the institutional guidelines for the Care and Use of Laboratory Animals and were approved by the Animal Ethics Committee of Nankai University (Approval No. 2024-SYDWLL-000023). To establish an orthotopic breast cancer model, 4T1 cells were inoculated into the mammary fat pad of each mouse. At a tumor volume of roughly 200 mm³, the mice were randomly allocated to the designated treatment groups. Then, mice were intravenously administered with 100 μL of nanoparticle solution containing 200 μg/mL ICG. The mice were then imaged at various time intervals using an IVIS system (Caliper Life Sciences) to monitor the *in vivo* fluorescence signals (Ex = 710 nm, Em = 800 nm).

The antitumor effect was evaluated in 4T1 tumor-bearing mice (tumor volume ~130 mm^3^) randomly divided into eight groups (n=6): PBS, ICG, PEG@ICN, cRGD/MD@ICN, each with or without laser irradiation. Mice were injected with ICG formulations (200 μg/mL, 100 μL, about 1 mg/kg). Four hours later, tumor of mice in each group received irradiation (808 nm, 0.5 W·cm^-2^, 5 min) every two days until four treatments were finished. After each irradiation, the tumor temperature was monitored by a thermal camera. Tumor size and body weight were recorded every day for 14 days to assess efficacy and systemic safety. Tumor volume was measured at daily intervals and calculated as follows: volume = (tumor length) × (tumor width)^2^/2.

**2.9 Inhibitory antitumor therapy for pulmonary metastasis *in vivo***

To establish a lung metastasis model, 4T1 cells were inoculated into the right abdominal mammary fat pad of each mouse to develop the primary tumor. Six days later, 4T1-luc cells (3×10^6^) were intravenously injected via the tail vein to induce pulmonary metastases. All subsequent experimental procedures were consistent with the protocol described previously. *In vivo* bioluminescence imaging was performed using an imaging spectroscopy system with a 60-second exposure time. Lungs were harvested and fixed in Bouin's solution for 20 h. The number of pulmonary micrometastases was counted across all five lung lobes, and the tissues were subsequently subjected to pathological analysis.

**2.10 DCs and T cells assessments**

After *in vivo* experiments, single-cell suspensions were prepared from the harvested tumors and spleens of mice. To analyze the infiltration of dendritic cells (DCs) and T lymphocytes in the tumor microenvironment and spleen, the suspensions were incubated with the following fluorescent-labeled antibodies: CD11c-BV421, MHCII-PerCP/Cy5.5, CD80-APC, CD86-PE, CD45-PE/Cy7, CD3-AF700, CD4-FITC, CD8-APC/Cy7 and CD69-BV650. Flow cytometry was subsequently performed to quantify immune cell populations, respectively. The gating strategies for DCs and T cells are presented in Figures S10 and S11, respectively. For DC analysis, cells were first gated on CD45^+^ (leukocytes), followed by CD11c^+^MHCII^+^ double-positive cells, and then CD80 and CD86 expression was analyzed within this population. For T-cell analysis, CD45^+^ cells were gated on CD3^+^, followed by CD4^+^or CD8^+^ subsets, and CD69 expression was analyzed within each subset. The main organs and tumors were excised and fixed in tissue fixative for further histochemical examination.

**2.11 Statistics analysis**

All quantitative data were obtained from at least three parallel experiments, and data are expressed as mean ± standard error of the mean (SEM). Statistical analyses were performed using GraphPad Prism. Comparisons among three or more groups were evaluated using one‑way analysis of variance (ANOVA). Significance levels are denoted as *p < 0.05, **p < 0.01, ***p < 0.001, and ****p < 0.0001.

**Figure S1.** MALDI-TOF analysis of DSPE-PEG2000-Mal and DSPE-PEG2000-MD.

**Figure S2.** ^1^H-NMR spectrum of DSPE-PEG2000-Mal and DSPE-PEG2000-MD in DMSO-d6.

**Figure S3.** Standard calibration curve of ICG in aqueous solutions (y=0.1912x - 0.0006, R^2^=0.9999).

**Table S1.** Encapsulation rate and drug loading rate of ICN under different ICG loading amount (n =3, Mean ± SD).

| ICG added (mg) | Encapsulation efficiency (%) | Loading efficiency (%) |
| --- | --- | --- |
| 1 | 98.93 ± 0.74 | 0.79 ± 0.02 |
| 2 | 90.57 ± 4.61 | 1.24 ± 0.20 |
| 4 | 82.01 ± 6.65 | 2.70 ± 0.35 |

**Figure S4.** UV absorption spectra of ICG formulations after laser irradiation for 5 min (808 nm, 0.5 W·cm^-2^).

**Figure S5.** Flow cytometric uptake analysis of 4T1 cells treated with (a-e) ICG samples (ICG concentration: 100/200 ng/mL, 2 h) or (c,f) cRGD/MD@ICN at different time points (n=3). (g) Lysosomal escape of cRGD/MD@ICN in 4T1 cells. Cells were incubated with cRGD/MD@ICN (red), followed by LysoTracker staining (green). Scale bar = 20 μm. *****p* < 0.0001, ****p* <0.001.

**Figure S6.** Cell viability of 4T1 cells after incubation with different concentrations of ICG and different nanoparticles for 24 h (n=3).

**Figure S7.** Fluorescence of DCFH-DA to indicate ROS generation of 4T1 cells by blank sample (control group) or different ICG samples containing 3 μg/mL ICG concentration. The notation "(+)" means laser irradiation (0.5 W·cm^-2^, 5 min).

**Figure S8.** H&E staining of organs from tumor-bearing mice after different treatments.

**Figure S9.** Plasma levels of biomarkers (ALT, AST, CRE, UA, and BUN) in 4T1 tumor-bearing BALB/c mice following with different treatments (n=3).

**Figure S10.** Representative gating strategies of flow cytometry analysis for dendritic cells (DCs).

**Figure S11.** Representative gating strategies of flow cytometry analysis for T cells.

**Figure S12.** Photographs of tumors in different groups after the anti-metastatic treatment was completed.
